# Control efficiency and mechanism of spinetoram seed-pelleting against the striped flea beetle *Phyllotreta striolata*

**DOI:** 10.1038/s41598-022-13325-8

**Published:** 2022-06-09

**Authors:** Xiong Tengfei, Satyabrata Nanda, Jin Fengliang, Lin Qingsheng, Feng Xia

**Affiliations:** 1 Institute of Plant Protection, Guangdong Academy of Agricultural Sciences, Guangzhou, China; 2 MS Swaminathan School of Agriculture, Centurion University of Technology and Management, Odisha, India; 3 Guangdong Provincial Key Laboratory of High Technology for Plant Protection, Guangzhou, China; 4 Engineering Research Center of Biological Control, Ministry of Education, South China Agricultural University, Guangzhou, China

**Keywords:** Entomology, Bioanalytical chemistry

## Abstract

The striped flea beetle (SFB, *Phyllotreta striolata*) can cause serious harm to cruciferous crops in both the larval and adult stages. Presently, there are no other sustainable alternatives to the use of chemical pesticides for controlling SFB infestation. In this study, the use of a seed-pelletized coating of spinetoram effectively reduced the numbers of SFB and its feedings on the flowering cabbage seedlings, whereas, in combination with the insect-proof net, it controlled the SFB infestation throughout the cabbage growth period. The analysis of the pesticide residues in soil and different cabbage parts indicated the degradation dynamics of spinetoram. The concentration of spinetoram in cabbage parts decreased over time, while increased first and subsequently decreased in soil. Furthermore, estimation of the half-life of spinetoram revealed that via seed-palletized application spinetoram half-life was found to be 2.82 days in soil, 4.21 days in the root, 5.77 days in the stem, and 3.57 days in the leaf, respectively. Both the lower pesticide residues and the half-life of spinetoram in soil and cabbage parts suggested it to be a promising environment and food-safe pesticide in controlling SFB. Moreover, the seed-pelletized coating ensured a sustainable release of spinetoram that can reduce the pesticide application frequency and be cost-effective and pocket-friendly for the farmers.

## Introduction

The striped flea beetle (SFB, *Phyllotreta striolata*) is a serious pest of cruciferous crops, including cabbages, broccoli, and radish^1,2^. Huge crop losses are seen in these crops in China due to the SFB infestations. Because of the surge in SFB infestation rates in China, it is considered to be the most dreadful pest of the cruciferous vegetables accounting for the crop loss of more than a billion US dollars^3–5^. SFB infestations can be devastating as both the larva and adults damage the crops by feeding on the leaves and stems of the plants. The feedings of adult SFBs cause holes on the leaf surface that result in decreased photosynthesis and leaf necrosis^6^. Furthermore, the larvae live in the soil and feed on the roots, which causes severe wilting and eventual death of the plants. As the SFBs complete their life cycles both underground and aboveground, the use of foliar sprays with insecticides and other chemicals does not ensure their irradiations^3,4^. The larvae can pupate in the soil and turn into adults via eclosion, and then can come onto the shoots and damage the crops^7^.

Conversely, the use of insecticides is the most practice method to control the of SFBs are also caused by the exploitation of insecticide usage in agricultural practices^8^. The SFB resistance against some prominent insecticides, including acephate, cypermethrin, and cyhalothrin has become even a greater problem in the prevention and control of SFB infestation (Zhou et al., 2004; Zhou et al., 2005). Furthermore, the use of multiple insecticides or their excessive uses can be detrimental to the environment^9^. Therefore, an effective and environment-friendly solution should be developed to manage the SFB infestations. In regard, the use of potent biopesticides, such as spinetoram can be a potential solution for SFB pest management.

Spinetoram is a natural-sourced environment-friendly biopesticide with high insecticidal efficiency and subtle on the natural enemies of SFB^10^. Spinosad and spinetoram can be degraded via a combination of photolysis and microbial action, ultimately producing CO_2_, H_2_O, and nitrogen oxides^11^. This suggests that along with a broad-pest spectrum crop applicability, spinosad and spinetoram are both environment- and human-friendly (Kai et al., 2018). Further, Crozier et al.^12^ reported that spinetoram did not adversely affect adult dogbane beetles when exposed to topical contact, but spinetoram caused high mortality to beetles when ingested. This result suggests that spinosad may have higher toxicity to herbivorous insects and lower toxicity to other insectivorous insects. It has already been used in the pest control of many crops and can be a promising candidate for SFB management^13,14^.

**Figure 1:**
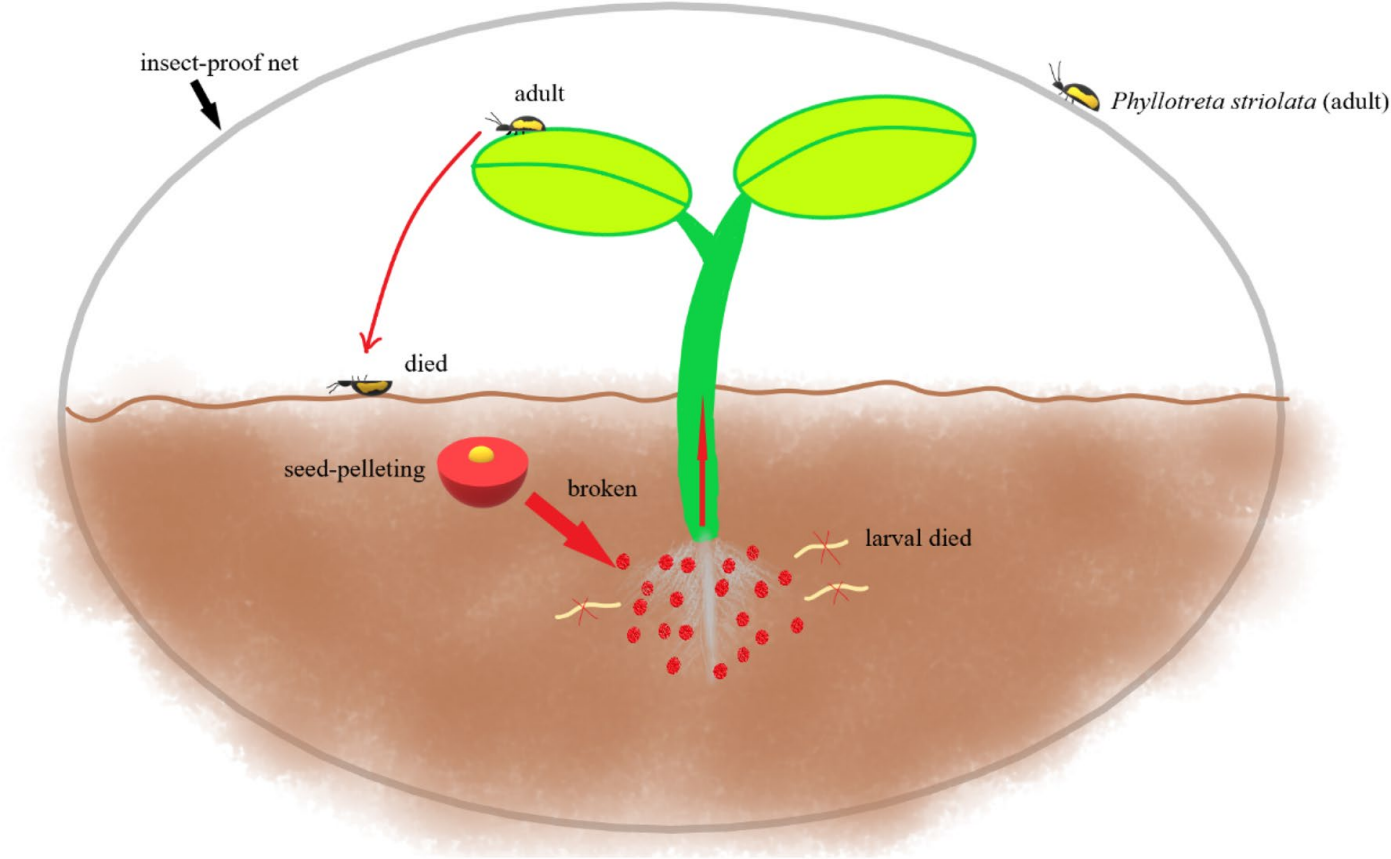
The use of a seed-pelletized coating of spinetoram effectively reduced SFB feedings on the flowering cabbage seedlings, whereas in combination with the insect-proof net, it controlled the SFB infestation throughout the cabbage growth period.

Seed pelleting and seed coating are two important techniques adopted in agricultural practice to achieve seed enhancements. In seed pelleting, the shape, size, and weight of a natural seed are altered by adding inert materials to it^15^. On the other hand, different substances, including chemicals, pesticides, fertilizers, and phytohormones are mixed with seeds in the process of seed coating to increase the seed performance. In this experiment, the dynamic trend of the residual amount of spinosad in the soil, increasing first and then decreasing, indicated that the seed pelletized material had a sustainable pesticide release effect. The sustainable release of pesticides helps to improve the utilization efficiency of pesticides^16^. Therefore, the seed-pelletized coatings can effectively increase the seed performance and can prevent them from different environmental stresses, including pest attacks^17,18^. Several studies have reported the influence of seed treatments in enhancing plant resistance against pathogens and pests. For instance, the use of fungicide seed treatments resulted in improved seed germination and broad-spectrum resistance against fungal pathogens in many field crops^19^. Similarly, seed treatments with imidacloprid were found to be the most effective in controlling leafhopper and thrip infestations in groundnut^20^. Further, the use of spinosad seed treatment alone or in combinations with other chemicals exhibited improved resistance against the onion maggots^21^. More recently, the farmer evaluations of the large-scale maize plantations that went through seed treatments with pesticides revealed that seed treatments are an effective means of controlling the fall armyworm infestations in maize (Chanda et al., 2021). In this study, we have evaluated the efficacy of the spinetoram seed-pelleting in controlling the SFB infestations in cabbage at the field level. Additionally, the lethal concentration 50 (LC_50_) of spinetoram against SFB was estimated. Further, we have analyzed the pesticide residues in the soil and in cabbage tissues to quantify the residual amounts. The degradation dynamics of the residual spinetoram in cabbage tissues were analyzed and the half-life of spinetoram was estimated. The findings of this study will add new and valuable insights into the use of spinetoram as a biopesticide and the spinetoram-based seed treatments in SFB management (Fig. 1).

## Results

### Effect of spinetoram seed treatments on SFB infestation and cabbage damage rates

**Figure 2:**
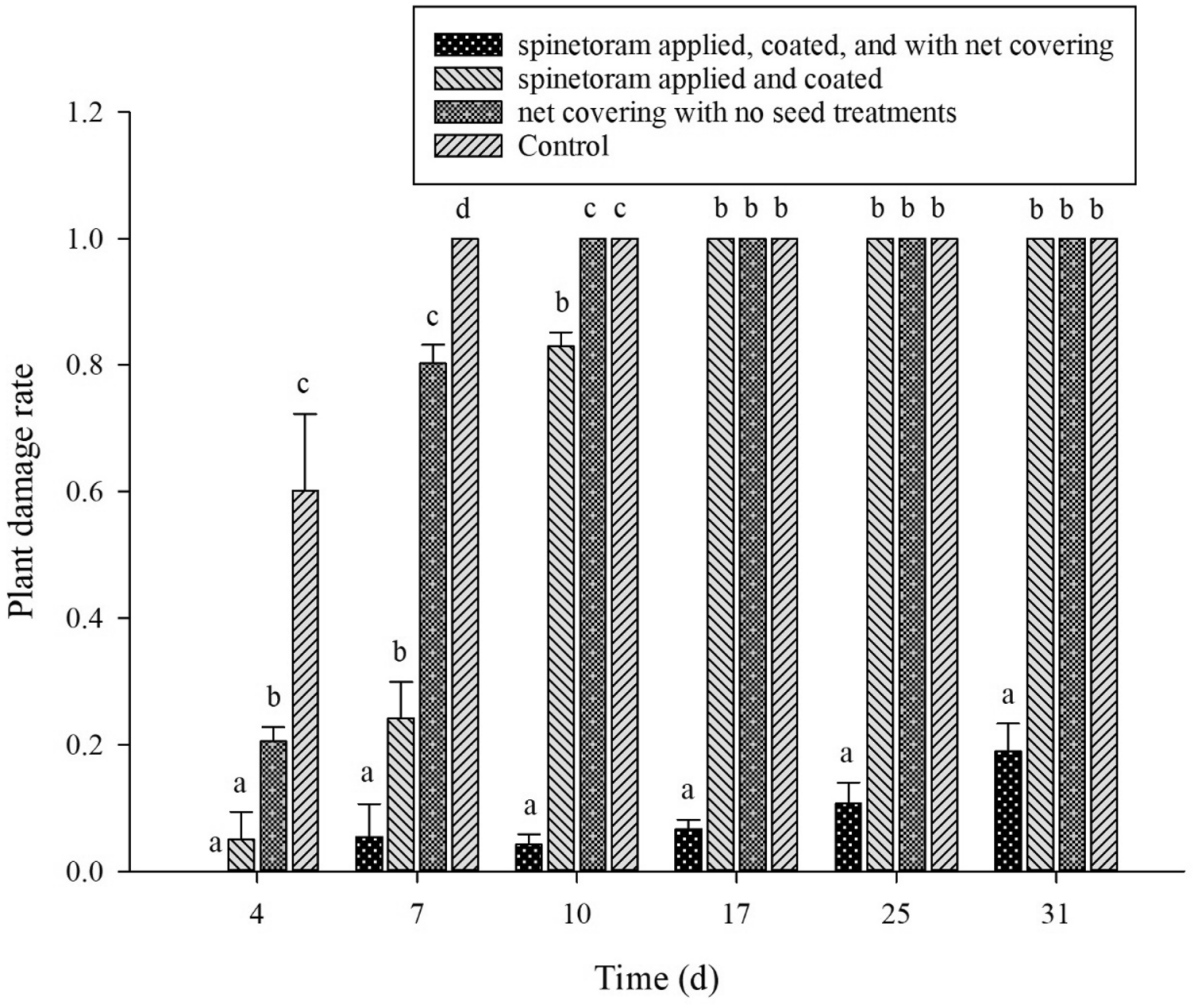
The damage rate on the flowering cabbage plants (group A–D) under control environment. The Y-axis represents the plant damage score and the X-axis represent the number of days post-treatment. Alphabets (a–c) indicate the statistical significance at *P* ≤ 0.05.

The effects of spinetoram seed treatments were evaluated in both controlled and field environments. In a controlled environment, the cabbage damage rates by SFB were found to be 0%, 5.08%, 20.50%, and 60.20%, respectively for group A (spinetoram applied, coated, and with net covering), group B (spinetoram applied and coated), group C (net covering with no seed treatments), and control (group D) on day 4. The damage rates were significantly higher in group C and group D as compared to group A or B (F = 87.59, *P* < 0.001). The damage rates were found to be increasing with the increase in infestation time (Fig. 2). On days 7 and 10 the damage rates were significantly higher in the control, group B, and group C compared with group A. On day 31, damage rates were significantly lower in group A as compared with other groups, which shared no significant difference among them (Fig. 2).

**Figure 3:**
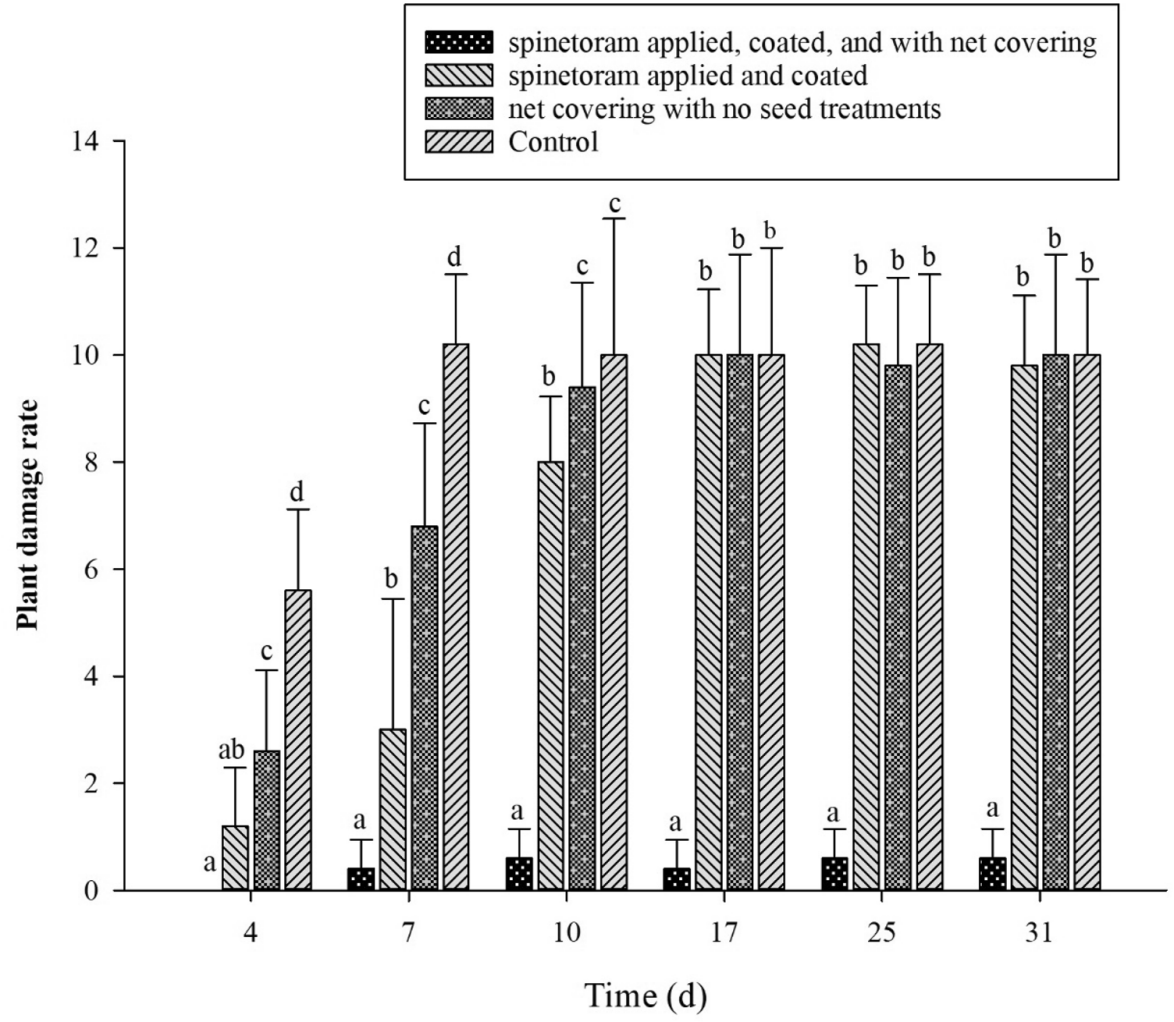
The damage rate on the flowering cabbage plants (group A–D) under field environment. The Y-axis represents the field population (SFB numbers) and the X-axis represents the number of days post-treatment. Alphabets (a–d) indicate the statistical significance at *P* ≤ 0.05.

On the other hand, the field environment studies revealed that on day 4, there were no significant differences found in-between groups A and B, and groups A and C among the field population of SFB. Conversely, a significant difference in the SFB numbers were found among groups C, B, and D (df = 3 F = 20.80 P < 0.001). On day 7, all groups were significantly different based on the infested SFB numbers on them. We have taken data from day 4 to day 31 on the filed evaluation. Interestingly, post-day 7 (from day 10 to day 31), there were no significant differences in the infested SFB numbers were observed among groups A, C, and D, whereas significant differences were found among groups B and A, C, D (Fig. 3).

### Degradation dynamics of the residual spinetoram in soil and different plant parts of flowering cabbage

**Figure 4:**
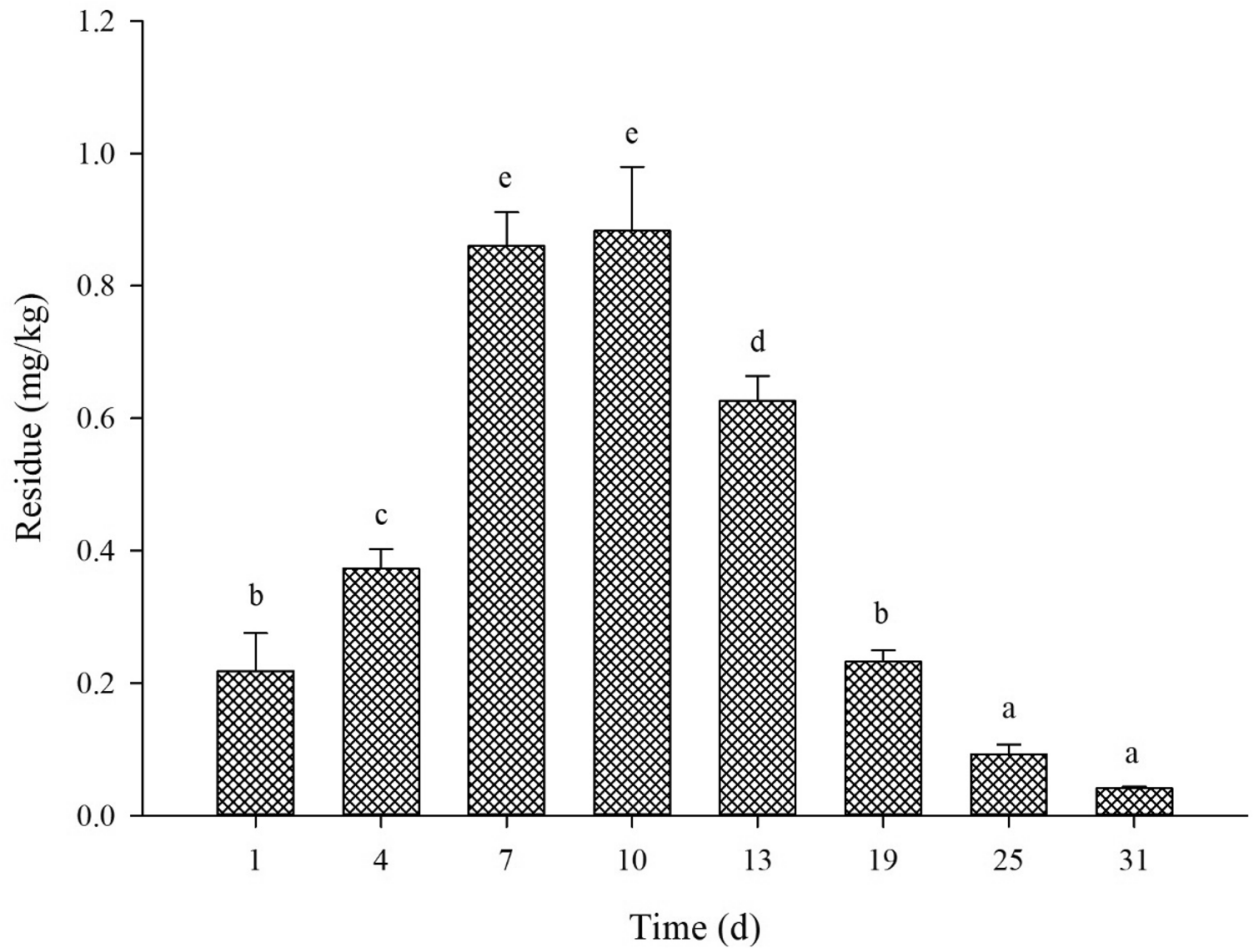
The degradation dynamics of spinetoram in the soil. The Y-axis represents the spinetoram residual amount and the X-axis represents the number of days post-treatment. Alphabets (a–d) indicate the statistical significance at *P* ≤ 0.05.

The spinetoram soil residues were estimated by using the external standard method, where the standard curve equation was deduced to be Y = 1501571X, with R^2^ = 0.9997, and a detection limit of 0.005 ppm. On days 1, 4, 7, 10, 13, 19, 25, and 31, the spinetoram residue was detected to be 0.22, 0.37, 0.86, 0.88, 0.63, 0.23, 0.09, 0.04 mg/kg, respectively in the soil. These results showed that the spinetoram residues were increasing in soil for the initial days of treatment, that is, from 0.22 to 0.88 mg/kg on day 1 to day 10. Subsequently, the spinetoram residual amounts started to decline gradually up to day 19, and then the amount declined sharply reaching 0.04 mg/kg on day 31 (Fig. 4).

**Figure 5:**
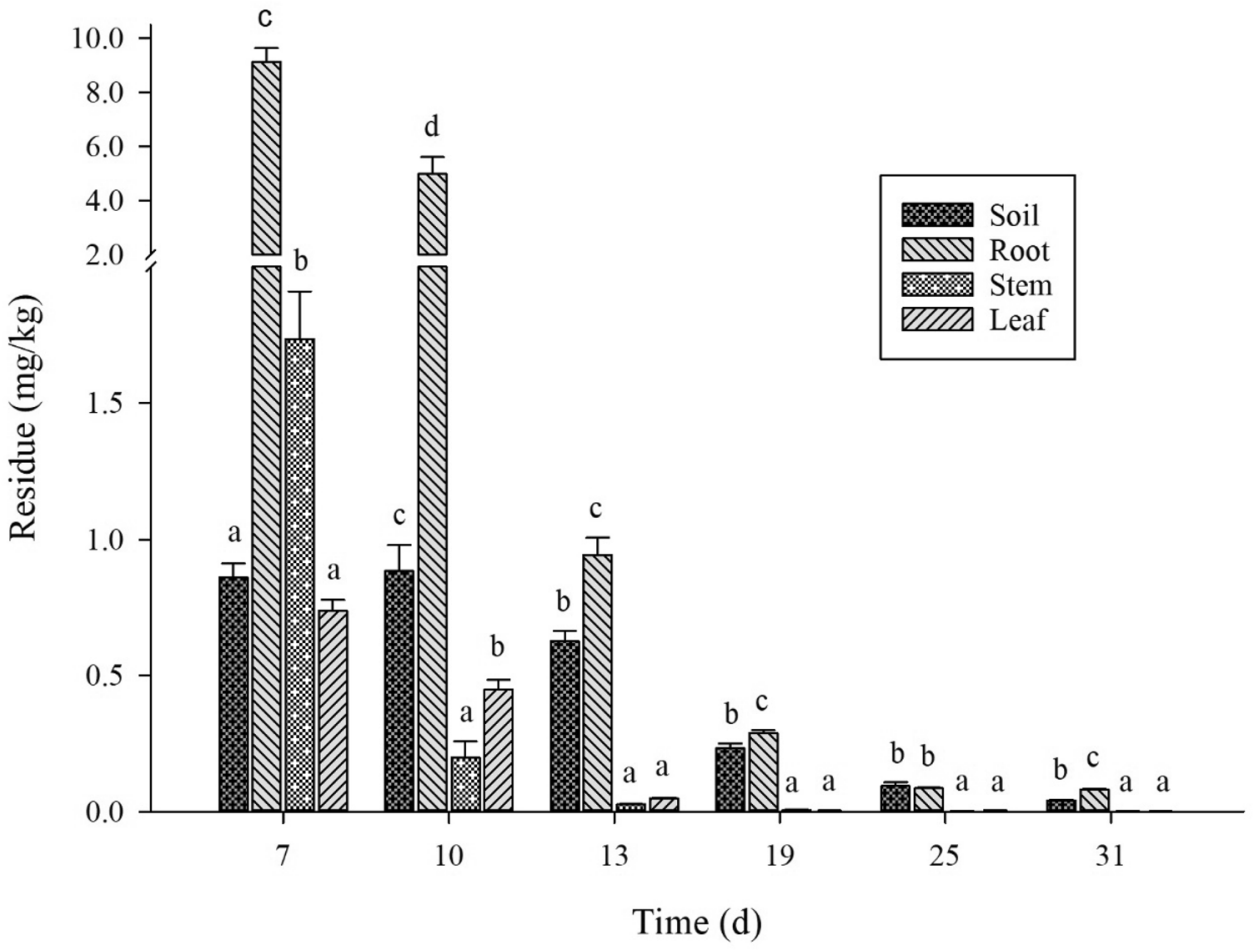
The degradation dynamics of spinetoram in different parts of the flowering cabbage. The Y-axis represents the spinetoram residual amount and the X-axis represents the number of days post-treatment. Alphabets (a–d) indicate the statistical significance at *P* ≤ 0.05.

A similar method of the spinetoram residues estimation was employed to evaluate the insecticide residue in the different plant parts of flowering cabbage. The results revealed that spinetoram residue in the roots to be 9.13, 4.97, 0.95, 0.29, 0.09, 0.08 mg/kg on days 7, 10, 13, 19, 25, and 31, respectively. Likewise, in stem the amount of spinetoram residue was estimated to be 1.74, 0.20, 0.03, 0.0051, 0.0023, and 0.0018 mg/kg on days 7, 10, 13, 19, 25, and 31, respectively. In leaves, the spinetoram residual concentration was found to be 0.74, 0.45, 0.05, 0.0039, 0.0037, and 0.0014 mg/kg on days 7, 10, 13, 19, 25, and 31, respectively. From the results, it is clear that the highest residual spinetoram was recorded in the roots as compared to other plant parts. Furthermore, the residual amounts decrease in all parts of the flowering cabbage with the progress in the days-post treatment (Fig. 5).

### Estimation of LC_50_ and half-life of spinetoram

Based on the different concentrations of spinetoram solutions (30 mg/L to 480 mg/L) and the respective SFB mortality rate the LC_50_ was estimated. The LC_50_ value was found to be 170.315 ppm (R^2^ = 0.951), while the toxic regression equation was derived to be Y = − 3.647 + 1.634X. On the other hand, the half-life of spinetoram was found to be different in soil and in different cabbage parts (Table 1). The half-life of spinetoram post-pelletized seed sowings in soil was found to be 2.82 days, while in cabbage roots, stem, and leaves were 4.21, 5.77, and 3.57 days, respectively.

**Table 1:** The half-life of spinetoram in the soil and different parts of the flowering cabbage.

Source	First order kinetic equation	R^2^	Rate constants	Half-life (in days)
Soil	C_t_ = 4.0485e^−0.1490t^	0.998	0.149	4.65
Root	C_t_ = 25.5993e^−0.2088t^	0.914	0.2088	3.32
Stem	C_t_ = 2.7959e^−0.2717t^	0.844	0.2717	2.55
Leaf	C_t_ = 3.0461e^−0.2718t^	0.886	0.2718	2.55

## Discussion

Agriculture in China is developing towards mechanization and informatization. Of the several adopted modern agricultural practices in China, the seed pelleting and coating is one^22^. Presently, several insecticides and biopesticides are used for seed coating to control the pest attacks on a variety of crops. Due to the high efficiency of pest control, lower toxicity, and environment-friendly nature, seed coating treatments with biopesticides are gaining popularity over the traditional chemical pesticide use on crops. Insecticides, such as neonicotinoids, carbaryl, and fipronil have already been used for seed treatments in the effective control of agricultural pests^23,24^. Further, many biopesticide seed treatments have been reported to enhance plant resistance against several pathogens and pests. For instance, treatment of *Bacillus subtilis* BY-2 in the seeds of oilseed rape enhanced the defense against the fungal pathogen *Sclerotinia sclerotiorum*^25^. Additionally, many fungal species, including *Beauveria bassiana, Paecilomyces fumosoroseus, Lagenidium giganteum*, and *Entomophaga maimaiga* are reported to have insecticidal properties and are used in seed treatments of several crops^26^. Likewise, spinosyn is a kind of biomolecule having insecticidal properties and is extracted from the gram-positive bacterial *Saccharopolyspora spinosa* after fermentation^27^. Spinetoram is the commercial form of spinosyns and can be used in seed treatments for effective pest control^26^. The use of spinetoram in protecting flowering cabbage against SFB infestations has already been reported^28,29^. In this study, we have evaluated the efficacy of spinetoram seed treatments in controlling the SFB infestations on flowering cabbage. In addition, we included a traditional pest protection system of net covering, both independently and along with spinetoram seed treatment to compare the pest control efficiency. The results revealed that the use of spinetoram seed treatment significantly reduced crop damage in the flowering cabbage plants under a controlled environment. However, under field evaluations, the crop damage by SFB was found to be significantly less in the spinetoram treated plants for the initial days (day 7), but later (day 10–31), the least damage was recorded on the crops treated with spinetoram and covered with a net (group B). A reason behind this could be the degradation of spinetoram in both soil and plant parts. Since, the growth period of flowering cabbage is 30–60 days, while spinetoram stays active only for 10–15 days, beyond that period plants are again exposed to the SFB infestation. Interestingly, among the three treatment groups in our research, the net covering with naked seeds (group C) had the worst prevention and treatment effect. Although the use of the net can prevent the SFB population outside from migrating inside the net, eggs and larvae of SFB in the soil can still damage crops within the nets. On the other hand, the pelletized seeds coated with spinetoram can effectively prevent the SFB from damaging flowering cabbage plants during the initial stage, but its prevention and control effect against SFB will be reduced with the decline in the concentration of spinetoram with time. Thus, we recommend that the use of both spinetoram seed-pelletize coating and insect-proof nets can effectively reduce the number of SFB population (including eggs and larvae) in vegetable fields and prevent the SFB population outside from settling on the plants, thereby protecting it throughout its life cycle. Spinetoram is a kind of insecticide friendly to natural enemy insects^30^, spinetoram seed treatments with an insect net can effectively reduce the chance of pesticide contact with natural enemy insects. Thus, spinetoram seed treatments can effectively control SFB and are safe for predators.

Insecticides with a short half-life are considered to be environment-friendly. The LC_50_ of spinetoram was estimated to be 170.315 ppm, categorizing it to be moderately toxic. Conversely, the half-life period of spinetoram was estimated to be less than 5 days in both soil and plants parts, indicating it to be environment friendly. In addition, at the pre-harvest stage, the spinetoram content in root, stem, and leaf was found to be 0.08 mg/kg, 0.0018 mg/kg, and 0.0014 mg/kg, respectively, meeting the relevant standards of pesticide residue. Thus, the method of spinetoram seed-pelletized coating can be considered in the future to be an environment and food-safe practice in agriculture. On the other hand, the seed-pelletized coating method has better efficiency in the sustained release of insecticide as compared with the traditional thin-film seed coating. In our findings, the concentration of spinetoram in soil showed a variation trend of increasing first and then decreasing, which could have been caused by the sustained release of seed-pelletized coating materials^31–33^. Thus, the use of a seed-pelletized coating of spinetoram can increase pesticide persistence, reduce pesticide application frequency, and lower the cost of application for the farmers.

In summary, our findings revealed that seed-pelletized coating of spinetoram can be a promising method of SFB control in cruciferous crops. The estimated LC_50_ and half-life values of spinetoram suggested that a small amount of spinetoram is needed for the effective control of SFB, whereas due to its rapid degradation in soil, a combination of seed treatment and use of an insect-proof net could be the best possible way to ensure the crop protection. Moreover, its short half-life period in soil and plant parts makes spinetoram an ideal eco-friendly and food-safe candidate for agricultural pest control. Finally, the sustained release of spinetoram from the pelletized and coated seeds can be cost-effective and pocket-friendly for farmer use.

## Materials and methods

### Insects and plants

The *P. striolata* (SFB) insect colonies were first obtained from the vegetable fields in Guangzhou, Guangdong Province, China. The insects were maintained in the laboratory at Guangdong Academy of Agricultural Sciences (GdAAS), Guangdong Province, China without any insecticide treatments for the last 2 years. Flowering cabbage (*Brassica oleracea*) was taken as the plant material for this study. Seeds of the flowering cabbage were obtained from GL seeds Ltd., China, and used for seed pelleting, treatment, and sowing in this study.

### Seed pelleting and treatment

A total of 50 g flowering cabbage seeds, 200 g of seed-pelleting materials (developed by Institute of Plant Protection, GdASS), 8.33 ml spinetoram suspension concentrate (60 g/L), and the required adhesives were added into the designated positions of the pelleting machine. Once the seed-pelleting was completed, the pelleted seeds were taken out and put in a drying machine for 40 min. Finally, the seed-pelletized and spinetoram coated seeds with a pesticide-seed proportion of 1:100 were obtained.

### Study design and field data collection

Four independent treatment groups were formed for this study: 1) spinetoram applied, coated, and with net covering (group A), spinetoram applied and coated (group B), net covering with no seed treatments (group C), conventional planting control (group D). The experiment was carried out with three replicates for each group. The random crossing method of sowing was adopted for all 12 experimental plots (4 groups × 3 replicates). On the 4th, 7th, 10th, 17th, 24th, and 31st day after sowing, a five-point sampling method was used for insect sampling to investigate the number of SFB adults, the number of flowering cabbages (damaged or undamaged), and the damage rate calculation^34^. The damage rates of the flowering cabbage plants were calculated as per the following formula:

$$\frac{\text{number of damaged flowering cabbage }}{\text{total number of flowering cabbage}} \times 100$$

### Estimation of the LC_50_ of spinetoram to SFB

Serial dilutions of spinetoram solutions, including 480 mg/L, 240 mg/L, 120 mg/L, 60 mg/L, and 30 mg/L were made. Leaf disks from the flowering cabbage leaves were produced by cutting with a cork borer. The leaf disks were immersed in the respective prepared spinetoram solutions for 10 s. Then, the disks were air-dried and put inside individual Petri dishes. Ten SFBs were added to each Petri dish and allowed to feed on the treated leaf disks. Leaf disks soaked in sterile water served as the control for this experiment. The mortality rates were checked at 48-h post SFB feedings and the data was recorded to calculate LC_50_. The experiment was performed having three independent replicates.

### Sample collection for pesticide residue analysis

For the pesticide (spinetoram) residue analysis both soil and plant samples were collected. For soil sampling, the soil around the roots of the treated cabbage plants (group A) from a depth of ≤ 5 cm was randomly collected on the 1st, 4th, 7th, 10th, 13th, 19th, 25th, and 31st days after sowing. The collected samples were immediately stored at − 20 °C until further use. For plant sampling, cabbage plant parts (group A), including roots, stem, and leaf were collected randomly and immediately stored at − 20 °C until further use.

### Pesticide residue detection

The pesticide residue detections were carried out by following the QuEChERS Method (Merck, Kenilworth, New Jersey, USA)^35^. Briefly, 10 ml of acetonitrile were added into the soil samples to disperse it evenly by vertexing. Then, it was subjected to ultrasonication and subsequent centrifugation, and the supernatant was collected. The supernatant was then purified by treatment of PSA, membrane filtered (0.22 μm), and then placed on the detector to estimate the pesticide residue. Similarly, to the plant samples, the corresponding amount of acetonitrile was added and soaked overnight. The soaked samples were then homogenized, centrifuged, and the supernatants were collected. The supernatant was then purified by treatment of PSA, membrane filtered (0.22 μm), and then placed on the detector to estimate the pesticide residue.

### Estimation of spinetoram dynamic degradation and half-life

The dynamic degradation of spinetoram was calculated by using the following formula:

$${C}_{T}={{C}_{0}}{e^{(-KT)}}$$

C_T_ is pesticide residue at the time of estimation; C_0_ is initial deposition after pesticide application; K is the degradation coefficient; T is time after pesticide application.

According to the above formula, the half-life of spinetoram was calculated based on:

$$\text{Half-life}\,{t}_{0.5}=18 \frac{ln2}{k}.$$

### Statistical analysis

The statistical significance of the experimental data was analyzed on SPSS Statistics 19.0. The one-way ANOVA, Duncan’s multiple range test (DMRT), Student’s t-test, and linear regression were used to analyze respective data sets as per their experimental design.

### Statement

The collection of experimental research and field studies on plants related to the article "Control efficiency and mechanism of spinetoram seed-pelleting against the striped flea beetle Phyllotreta striolata" complies with the relevant laws of Mainland China.
